# CrLHP1-CrJAZ1 Module Regulates Monoterpenoid Indole Alkaloid Biosynthesis via JA Signaling in *Catharanthus roseus*

**DOI:** 10.3390/genes17050569

**Published:** 2026-05-17

**Authors:** Bingrun Yang, Wenhui Ma, Jianing Cheng, Xiaoxiao Gao, Fang Yu

**Affiliations:** 1 School of Biological Engineering, Dalian Polytechnic University, Dalian 116034, China; yangbr185855@163.com (B.Y.); mwh24630@163.com (W.M.); 2 College of Bioscience and Biotechnology, Shenyang Agricultural University, Shenyang 110866, China; chengjianing@syau.edu.cn

**Keywords:** *Catharanthus roseus*, *CrLHP1*, monoterpene indole alkaloids biosynthesis, jasmonic acid signaling, CrJAZ1

## Abstract

**Background/Objectives:** Epigenetic regulation plays a fundamental role in controlling the spatiotemporal expression of genes in plants under stressful environmental conditions. While LIKE HETEROCHROMATIN PROTEIN 1 (LHP1) is known to be involved in histone modification, its function in regulating the biosynthesis of specialized metabolites, particularly monoterpenoid indole alkaloids (MIAs) in *Catharanthus roseus*, remains elusive. **Methods:** CrLHP1 was identified by mining the *C. roseus* proteome and characterized through sequence alignment, phylogenetic analysis, and conserved domain assessment. Virus-induced gene silencing (VIGS) was employed to suppress *CrLHP1* expression, after which the transcript levels of jasmonic acid (JA)-responsive genes and key MIA biosynthetic genes, as well as the accumulation of vindoline and catharanthine, were analyzed. Furthermore, deep learning-based protein structure prediction (AlphaFold3) and yeast two-hybrid (Y2H) assays were conducted to explore protein-protein interactions. **Results:** CrLHP1 was confirmed as the ortholog of *Arabidopsis thaliana* LHP1 (AtLHP1). Exposure to 75 μM MeJA upregulated MIA upstream pathway genes while downregulating *CrLHP1* transcription. Silencing *CrLHP1* significantly upregulated JA-responsive and MIA biosynthetic genes, leading to enhanced catharanthine accumulation. Additionally, the structural prediction and Y2H assays revealed a physical interaction between CrLHP1 and CrJAZ1. **Conclusions:** These findings suggest that CrLHP1 negatively regulates MIA biosynthesis, potentially by modulating JA signal transduction through interaction with CrJAZ1. This study provides new insights into the possible epigenetic mechanisms governing alkaloid production in *C. roseus*.

## 1. Introduction

*C*. *roseus* (L.) G. Don, a taxonomically significant member of the Apocynaceae family, is regarded as a medicinal model species due to its unique metabolic repertoire. This plant serves as the sole natural source for the clinically irreplaceable antineoplastic bisindole alkaloids, vincristine and vinblastine, which remain foundational to the chemotherapeutic management of Hodgkin’s lymphoma and various pediatric leukemias [1,2]. The biosynthesis of these high-value dimeric alkaloids proceeds through the stereospecific enzymatic coupling of two monomeric monoterpenoid indole alkaloids (MIAs), specifically the indole-type precursor catharanthine and the dihydroindole-type unit vindoline [3,4]. Despite their profound pharmacological necessity, the industrial-scale availability of these therapeutic agents is critically bottlenecked by their trace-level *in planta* accumulation, which typically accounts for less than 0.001% of the total tissue dry weight [5]. Furthermore, the high structural and stereochemical complexity of these alkaloids renders total chemical synthesis an economically prohibitive alternative for commercial production. Consequently, a characterization of the complex regulatory networks orchestrating MIA biosynthesis represents a fundamental imperative for advancing metabolic engineering and synthetic biology initiatives directed toward augmenting alkaloid productivity to meet global clinical demands [6].

In *C. roseus*, MIA biosynthesis is governed by a complex signaling network, within which the jasmonate (JA) pathway functions as a master regulator [7]. The central significance of JA stems from its dual role in modulating intrinsic developmental processes and orchestrating adaptive responses to environmental challenges [8,9]. Following the perception of external stimuli, ranging from herbivory and pathogen infection to abiotic stressors such as mechanical wounding, plants rapidly trigger JA biosynthesis as a core defense mechanism [10].

To ensure a robust defense while preventing detrimental resource exhaustion, the JA signaling pathway is tightly modulated via multiple layers of negative feedback [11]. In the model species *A. thaliana*, the JASMONATE ZIM-DOMAIN (JAZ) protein family acts as the primary repressor of the JA cascade [12]. Under homeostatic conditions with basal jasmonoyl-isoleucine (JA-Ile) levels, JAZ proteins sequester and inhibit key JA-responsive transcription factors, thereby maintaining the pathway in a repressed state [13]. Conversely, upon JA-Ile perception, the F-box protein CORONATINE INSENSITIVE 1 (COI1) functions as a co-receptor to assemble the SCF-COI1 E3 ubiquitin ligase complex, which facilitates the polyubiquitination and subsequent 26S proteasome-mediated degradation of JAZ repressors [14,15]. This derepression mechanism liberates TFs, such as members of the MYC2 family, enabling them to bind promoter regions and initiate the expression of downstream genes involved in defense and specialized metabolism [16].

Emerging evidence suggests that JAZ-mediated repression extends significantly beyond simple direct physical inhibition of transcription factors [17,18]. These proteins are increasingly recognized as versatile scaffolding platforms that recruit specific epigenetic modifiers to enforce a stable and heritable “off” state of chromatin. In *Arabidopsis*, JAZ proteins have been shown to physically associate with LIKE HETEROCHROMATIN PROTEIN 1 (LHP1) [19], which constitutes a hallmark component of the Polycomb Group (PcG) protein complex [20]. LHP1 operates as a specialized molecular reader of the H3K27me3 (histone H3 lysine 27 trimethylation) repressive mark to promote localized chromatin condensation and gene silencing [21,22,23]. In recent years, epigenetic regulation has been widely recognized as a crucial layer of control governing plant specialized metabolism [24,25]. However, while the transcriptional cascades regulating MIA biosynthesis in *C. roseus* have been extensively characterized, the specific epigenetic mechanisms orchestrating these pathways remain largely elusive. While the JAZ-LHP1 module is recognized as a critical molecular brake for the growth-defense trade-off in model plants, its existence and functional contribution to the specialized metabolism of high-value medicinal plants like *C. roseus* remain largely unexplored.

Specifically, whether this integrated epigenetic-transcriptional module acts as a primary constraint on the MIA biosynthetic pathway constitutes a fundamental knowledge gap. Therefore, this study aims to identify the *LHP* gene family in *C. roseus*, elucidate its interplay with the JA signaling cascade, and characterize its regulatory impact on MIA biosynthesis, thereby addressing a critical gap regarding the higher-order regulation of specialized metabolism. This research provides mechanistic evidence of an epigenetic layer in JA-mediated MIA regulation and offers a novel biotechnological strategy for the targeted enhancement of high-value alkaloid production in medicinal plants.

## 2. Materials and Methods

### 2.1. Identification of LHP1 Homologs in C. roseus

To identify potential LHP1 homolog sequences within the *C. roseus* genome, this study employed a search strategy based on a Hidden Markov Model (HMM), implemented using HMMER v3.3.2 [26]. The standard HMM profile corresponding to the highly conserved N-terminal chromodomain (PF00385) of *A. thaliana* LHP1 protein was retrieved from the Pfam database of protein domain functional annotations. Using this model, the hmmscan algorithm from the HMMER suite was executed. This algorithm performed a scan alignment against the entire *C. roseus* proteome (NCBI RefSeq assembly accession: GCA_024505715.1), applying a predefined significance threshold (E-value ≤ 1 × 10^−2^).

### 2.2. Sequence Alignment and Phylogenetic Analysis of LHP1 Orthologs

Multiple sequence alignment of LHP orthologs from *C. roseus*, *A. thaliana*, *Solanum lycopersicum*, and *Oryza sativa* was performed with MAFFT v6.864 (default parameters). The final alignment results were visualized using the ESPript 3.2 online platform, employing the default color scheme to label conserved regions [27]. To assess phylogenetic relationships, trees were constructed using the Maximum Likelihood method implemented in MEGA 11. The best-fit substitution model (LG + G) was selected according to the Bayesian Information Criterion (BIC). Branch support was evaluated using 1000 bootstrap replicates. Gaps and missing data were treated with partial deletion (95% site coverage cutoff). The Nearest-Neighbor-Interchange (NNI) heuristic search was used, with an initial tree generated by the Neighbor-Joining method. The putative *CrLHP1* gene in *C. roseus* was preliminarily identified as the ortholog that clustered with the *A. thaliana*, *S. lycopersicum*, and *O. sativa* orthologs on branches with the shortest evolutionary distances.

### 2.3. Conserved Domain Identification and Gene Structure Visualization

To elucidate the evolutionary characteristics and structural diversity of the target gene family, this study integrated analyses of conserved domains and gene structures. First, conserved motifs were identified using MEME Suite (v5.5.9) with parameters set as follows: maximum number of motifs = 10, motif width range = 6–50 amino acids (aa), and significance cutoff E-value < 1 × 10^−10^ [28]. Subsequently, based on genomic annotation files, gene structure schematics were generated employing the “Gene Structure View (Advanced)” feature within TBtools v2.39 [29]. These schematics visualize exon-intron organization and relative positions. By overlaying positional information of conserved motifs (denoted by distinct colors), the correlation between protein domains and gene architectures was elucidated. Finally, TBtools was used to integrate and output high-resolution vector graphics, visually representing the structural conservation and diversification patterns within the gene family.

### 2.4. Plant Materials and Cultivation

*C. roseus* (L.) G. Don seeds were surface-sterilized and germinated on sterile filter paper for 10 days under moist conditions. Germinated seedlings were then transferred to soil-filled pots. Plants were maintained for an additional 3 weeks in a controlled greenhouse at 26/18 °C (day/night), 60% relative humidity, and a 16-h photoperiod. The growth environment was maintained at a PAR level of 1500 ± 50 μmol m^−2^ s^−1^ using supplemental plant grow lamps positioned 5 cm above the plants in the greenhouse.

### 2.5. Total RNA Extraction and Molecular Cloning of CrLHP1

To clone the target gene *CrLHP1*, total RNA was extracted from freshly collected *C. roseus* leaves using TRIzol reagent (Sangon Biotech (Shanghai, China) Co., Ltd.) according to the manufacturer’s protocol. Fresh leaves were flash-frozen in liquid nitrogen and ground to a fine powder prior to RNA isolation [2]. The extracted total RNA served as template for first-strand cDNA synthesis using M-MLV reverse transcriptase (TaKaRa Bio, Beijing, China). This cDNA was then used as template for PCR amplification of the *CrLHP1* gene fragment using gene-specific primers (*CrLHP1*-F and *CrLHP1*-R sequences in Supplementary Table S1) and PrimeSTAR Max DNA polymerase (TaKaRa Bio, Beijing, China). The resulting PCR amplicon was gel-purified and subsequently subjected to A-tailing using ExTaq DNA polymerase (TaKaRa). The A-tailed fragment was then cloned into the pGEM-T Easy vector (Promega Corporation, Beijing, China) to generate the recombinant plasmid, designated T-*CrLHP1*.

### 2.6. Virus-Induced Gene Silencing (VIGS) of CrLHP1 in C. roseus

For VIGS vector construction, 302 bp fragment of *CrLHP1* was PCR-amplified and then cloned into the pTRV2 vector with different cloning sites (*Bam*H I/*Kpn* I) to produce silencing VIGS vectors. The primer sequences and cloning sites used for VIGS vector construction are listed in Table S1. The empty and constructed VIGS vectors (pTRV2, pTRV2-*CrLHP1*, and pTRV1) were then transformed into *Agrobacterium tumefaciens* GV3101, and VIGS treatments for silencing the target gene were performed according to the method developed previously by Gao et al. [30]. *A. tumefaciens* cultures were grown in LB medium (50 µg/mL kanamycin, 10 mM MES, 20 µM acetosyringone) at 28 °C overnight, harvested, and resuspended in infiltration buffer (10 mM MgCl_2_, 20 mM MES, 200 µM acetosyringone) for a 3- h incubation at 28 °C. The stem of 4-wk-old plants just below the meristem was wounded with a syringe needle containing the *A. tumefaciens* suspension. The VIGS-treated plants were cultivated in a growth chamber under standard conditions. Three weeks post-infection, newly emerged leaf pairs (1 & 2 from the top) were harvested for gene expression and MIA analysis [5]. Each biological replicate consisted of leaves collected from an independent VIGS-treated plant at the same developmental age.

### 2.7. Gene Expression Analysis

To investigate the effects of *CrLHP1* silencing on the JA signaling and MIAs biosynthesis gene expression in *C. roseus* leaves, total RNA from leaves was isolated from each treatment using TRIzol Reagent according to the manufacturer’s instructions [2]. The extracted total RNA was reverse-transcribed to cDNA using a reverse transcription kit for real-time PCR analysis. Real-time PCR was performed using SYBR Green Supermix, 5 μM of primers, and cDNA templates (equivalent to 5 ng total RNA) in a 20 μL reaction volume. The forward and reverse primer sequences are listed in Table S2. The real-time PCR was conducted under the following conditions: 95 °C for 1 min, followed by 40 cycles of 95 °C for 20 s, 50 °C for 15 s, and 72 °C for 15 s. The relative gene expression levels were determined using the 2^−ΔΔCt^ method and were normalized to *CrActin1*.

### 2.8. HPLC Analysis

Sample preparation followed a published protocol [30]. Young *C. roseus* leaves were dried at 45 °C for 48 h. Subsequently, 0.2 g of the dried material was extracted with methanol (1.0 mL) for 1 h to isolate total terpenoid indole alkaloids (MIAs). The extracts were centrifuged at 10,000× *g* (approximately 12,000 rpm) for 10 min. The clarified supernatant was then filtered through a 0.22 µm membrane. Vindoline and catharanthine were quantified via high-performance liquid chromatography (HPLC) using an Exformma EX1800 instrument (Wufeng Scientific Instruments Co., Ltd., Shanghai, China) maintained at 25 °C. Separation was achieved on a C18 column (250 mm × 4.6 mm, 5 μm particle size). An isocratic mobile phase of methanol and water (60:40, *v*/*v*) was applied at a flow rate of 1.0 mL/min. Certified vindoline and catharanthine standards (Sigma-Aldrich, St. Louis, MO, USA) were used to construct external calibration curves. The eluted peaks were detected using a diode array detector (DAD) at 310 nm for vindoline and 280 nm for catharanthine.

### 2.9. Protein–Protein Interaction Structure Prediction

AlphaFold Server (https://alphafoldserver.com/) was used to predict the three-dimensional structures of protein complexes. The full-length amino acid sequences of proteins CrJAZ1 and CrLHP1 were submitted to the server in the specified order, using default multimer prediction settings. Models were generated by integrating coevolutionary signals with physical structural constraints through deep learning methods. For each complex, five predicted models were output, accompanied by local confidence (pLDDT). The model with the highest confidence (ipTM + pTM > 0.8) was selected as the representative structure for subsequent analysis. All predicted results are explicitly designated as “predicted models” to distinguish them from experimentally determined structures. The CrJAZ1-CrLHP1 interaction interface coloring scheme is based on AlphaFold’s predicted pLDDT values, indicating per-residue confidence: dark blue (pLDDT > 90, very high confidence), light blue (90 > pLDDT > 70, high confidence), yellow (70 > pLDDT > 50, medium confidence), orange (pLDDT < 50, low confidence). Residues within the predicted interaction interface predominantly displayed medium to high confidence (light blue/yellow).

### 2.10. Yeast Two-Hybrid Assay

To investigate the interaction between LHP1 and JAZ1 in *C. roseus*, the coding regions of *CrJAZ1* and *CrLHP1* were fused to BD (binding domain) and AD (activation domain) and cloned into the pGBKT7 and pGADT7 vectors, respectively, using standard molecular cloning techniques via *Nde* I and *Bam*H I restriction sites. The recombinant plasmids were co-transformed into *Saccharomyces cerevisiae* strain AH109 via the lithium acetate/polyethylene glycol (LiAc/PEG) method. Co-transformants were selected on Synthetic Defined (SD) agar medium lacking tryptophan and leucine (SD/-Trp/-Leu) to select for retention of both plasmids. Plates were incubated at 30 °C for 3–5 days. Selected colonies were patched or streaked onto SD/-Trp/-Leu/-His and SD/-Trp/-Leu/-His/-Ade at 30 °C for 3–7 days. Co-transformants of pGBKT7-53 and pGADT7-T were used as positive controls, while pGBKT7 and pGADT7 were used as negative controls.

### 2.11. Statistical Analysis

Statistical analyses were performed using SPSS 20.0 software, and significance was determined via Student’s *t*-tests (*: *p* < 0.05, **: *p* < 0.01).

## 3. Results

### 3.1. Genomic Localization and Structural Characterization of LHP1 in C. roseus

**Figure 1:**
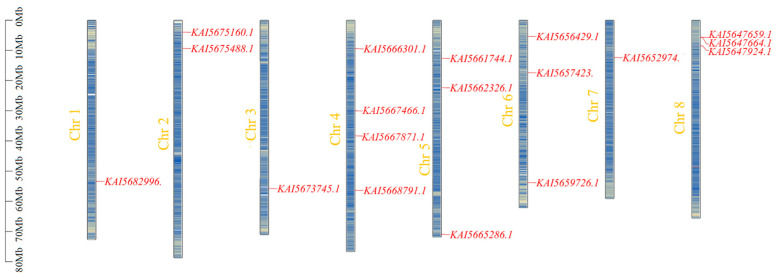
Distribution of LHP gene family members on *C. roseus* chromosomes. Each vertical bar represents a chromosome, numbered on the left side. Short red lines indicate the relative position of each *CrLHP* gene. The numbered axis shows the physical scale (in megabases, Mb). This figure was generated using genomic data from *C. roseus* (NCBI RefSeq assembly: GCA_024505715.1).

HMMER scans targeting the conserved Chromodomain (PF00385) identified 18 putative *LHP* genes within the *C. roseus* genome. Physical mapping revealed an uneven distribution of these genes across all 8 chromosomes. Chromosome 4 harbors the highest number of genes (4) distributed across dispersed loci, whereas Chromosomes 1, 3, and 7 contain only 1 gene each. Notably, a localized high-density cluster comprising 3 members (*KAI5647659.1*, *KAI5647664.1*, and *KAI5647924.1*) was observed at the proximal end of Chromosome 8 (Figure 1). This clustered arrangement indicates that tandem duplication events likely played a critical role in the local expansion of this gene family.

**Figure 2:**
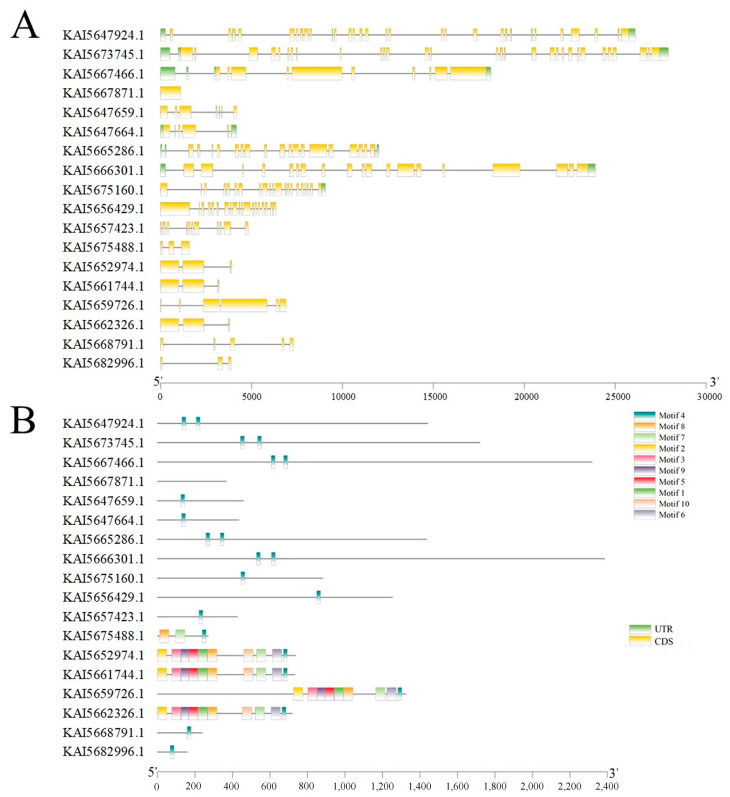
Gene structures and conserved protein domains of the *C. roseus LHP* gene family. (**A**) Schematic representation of the gene structures of *CrLHP* gene. Exons are depicted as yellow rectangles, introns as black lines, and the 5′/3′ untranslated regions (UTRs) as green boxes. Gene lengths are proportional; the scale bar indicates base pairs (bp). (**B**) Analysis of conserved domains within the CrLHP proteins. Key domains identified using the NCBI CDD database include: a Chromodomain (blue) present in all members.

Furthermore, these members exhibited massive structural heterogeneity in their genomic organizations. Exon numbers fluctuated from 1 to 30, and intron lengths spanned 0.2 kb to 3.2 kb, resulting in overall gene lengths approaching 30 kb in some members such as *KAI5647924.1* and *KAI5673745.1* (Figure 2A). At the protein level, phylogenetic and motif composition analyses elucidated their evolutionary divergence and revealed two distinct patterns in protein architectures. Although all candidates possess the characteristic Motif 4 representing the canonical chromodomain essential for chromatin binding, most members exhibit a simplified architecture restricted predominantly to this core motif (Figure 2B).

In contrast to the tandemly duplicated gene cluster that possesses this simplified structure, the specific evolutionary clade comprising 4 members, *KAI5652974.1*, *KAI5661744.1*, *KAI5659726.1*, and *KAI5662326.1*, displays a highly complex architecture harboring up to 10 distinct motifs (Figure 2B). Taken together, these genomic and protein structural divergences suggest that the *LHP* gene family underwent substantial evolutionary diversification and potential functional divergence following historical gene duplication events.

### 3.2. Phylogenetic Analysis and Cloning of the Core Homolog CrLHP1

**Figure 3:**
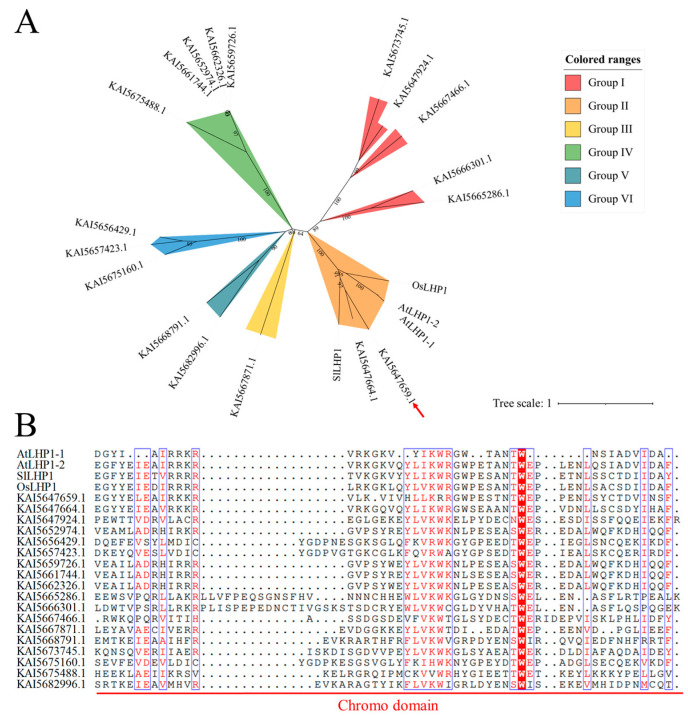
Phylogenetic analysis and multiple sequence alignment of LHP1 orthologs in *C. roseus* and *A. thaliana*. (**A**) The phylogenetic tree was constructed using the maximum likelihood method implemented in MEGA 11. The scale bar denotes the unit evolutionary distance, reflecting the number of substitutions per site accumulated along each branch. (**B**) Analysis of LHP protein domain sequence alignment. Alignment results revealed conserved sequence features within the Chromodomain of LHP proteins from *A. thaliana*, *S. lycopersicum* and *O. sativa* and *C. roseus*, as indicated by the red line.

Given the pivotal regulatory functions established for AtLHP1, we sought to identify its functional orthologs in *C. roseus*. Phylogenetic analysis revealed that two *C. roseus* protein candidates, KAI5647659.1 and KAI5647664.1, cluster tightly with AtLHP1 within the same evolutionary clade (Group II), suggesting they are conserved orthologs (Figure 3A). Furthermore, multiple sequence alignment confirmed a high degree of structural conservation within the characteristic Chromodomain among these sequences (Figure 3B). While both candidates share significant sequence identity, Pearson correlation analysis of transcriptomic data demonstrated that *KAI5647659.1* exhibits a significantly higher correlation with MIA biosynthetic genes than *KAI5647664.1* (Figure S1). Based on its robust transcriptional profile and structural conservation, we selected KAI5647659.1 as the primary target for further characterization and designated it as CrLHP1. To validate these bioinformatic predictions and facilitate subsequent functional analyses, we amplified the full-length open reading frame of *CrLHP1* via PCR (Figure S2).

### 3.3. The Effects of Exogenous MeJA on CrLHP1 and Key MIAs Biosynthetic Gene Expression in C. roseus

**Figure 4:**
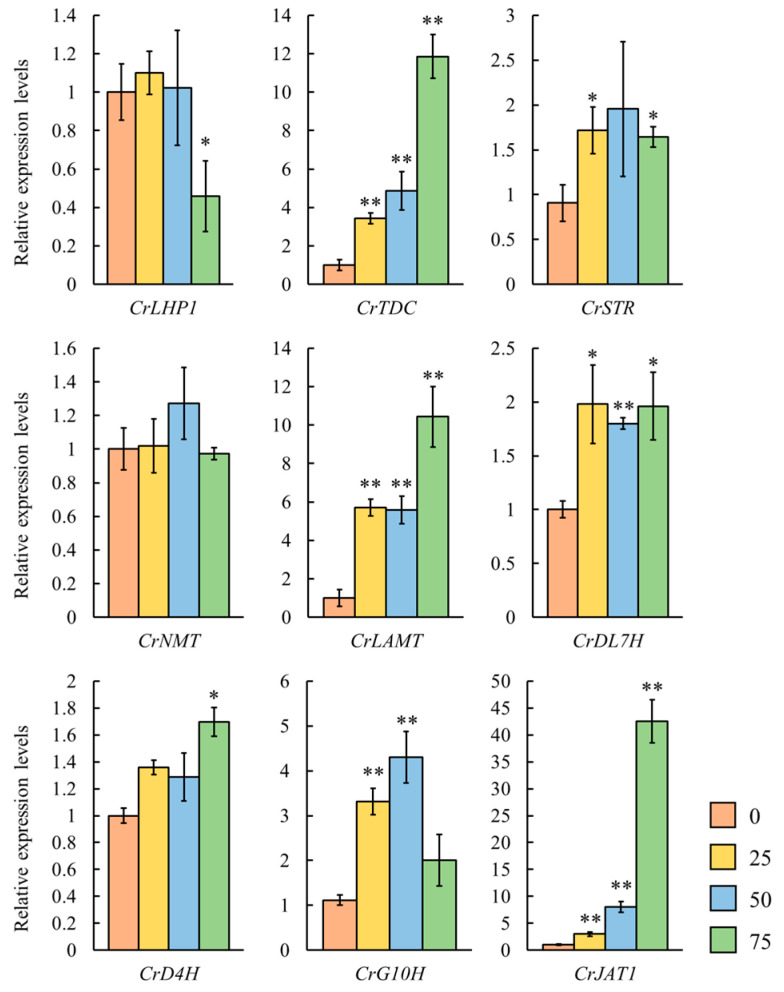
Effect of exogenous JA on *CrLHP1* and key MIA biosynthetic genes in *C. roseus* leaves. The qRT-PCR results were normalized to *CrActin1* expression and expressed relative to the baseline level in solvent-treated leaves. *CrJAT1*, a JA signaling marker gene, served as the positive control. Sterile *C. roseus* seedlings (with one pair of true leaves) were pre-incubated in liquid half-strength MS medium (1/2 MS salts supplemented with 15 g/L sucrose) for 1 day at 25 °C with shaking. MeJA (prepared as a 1000× stock solution in DMSO) was then added to the medium to give final concentrations of 0, 25, 50, and 75 µM for 2 h treatments. Standard errors, calculated from 3 independent biological replicates (each consisting of 10 leaves collected from 10 plants of similar size and developmental stage), are denoted by error bars. Statistical significance was determined using Student’s *t*-test (*: *p* < 0.05, **: *p* < 0.01).

Building upon the well-documented inductive effect of exogenous MeJA on MIA biosynthesis in *C. roseus* [5], we investigated whether CrLHP1 mediates JA signaling by treating leaves with a MeJA concentration gradient of 0, 25, 50, and 75 μM for a duration of 2 h. Treatment with 75 μM MeJA significantly (*p* < 0.05) upregulated key enzyme genes governing early and intermediate steps of MIA biosynthesis (*CrTDC*, *CrSTR*, *CrG10H*, *CrDL7H*, and *CrLAMT*), indicating that short-term exposure to high-concentration MeJA specifically activates the upstream section of the MIA pathway (Figure 4). Conversely, the transcript levels of *CrD4H* and *CrNMT*, which encode enzymes for the late modification steps of MIA biosynthesis, showed no significant differences relative to the control across all MeJA concentrations after 2 h of treatment (*p* > 0.05). This suggests that JA signaling regulates MIA biosynthesis, primarily by targeting upstream indole and iridoid pathway genes.

Notably, *CrLHP1* expression was significantly downregulated (*p* < 0.05) following 75 μM MeJA treatment. This contrasting inhibitory trend of *CrLHP1* sharply differed from the induction observed for most MIA biosynthetic genes. Therefore, MeJA exerts stage-specific regulatory effects on the MIA biosynthetic pathway in *C. roseus*, potentially mediated through modulation of *CrLHP1* expression. This finding supports the hypothesis that CrLHP1 regulates MIA biosynthesis by acting within the JA signaling pathway.

### 3.4. Silencing of CrLHP1 Modulates JA Signaling and Promotes Catharanthine Accumulation

**Figure 5:**
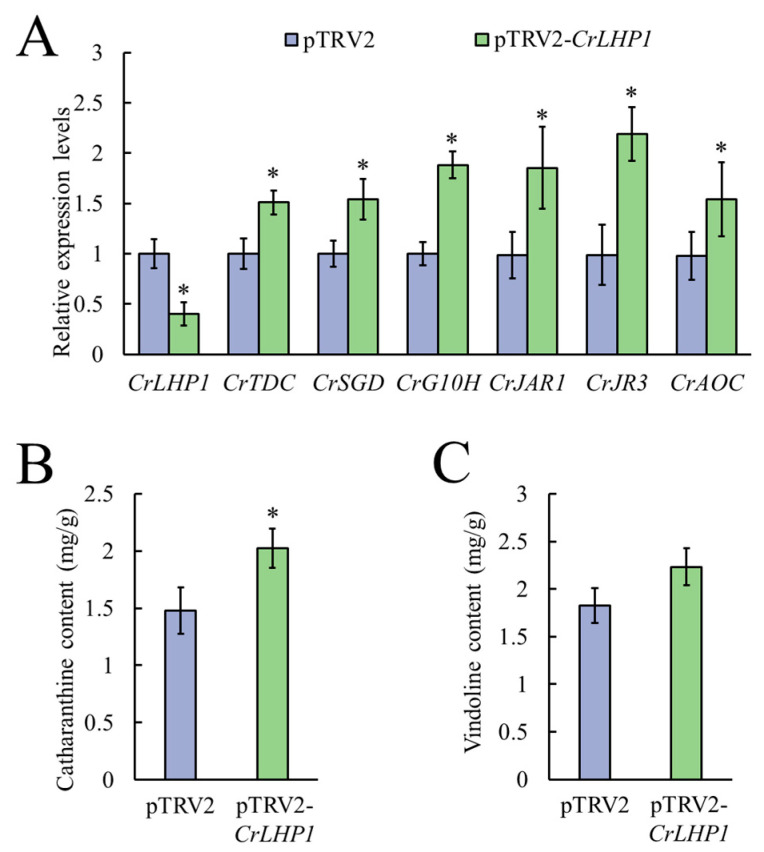
Effect of *CrLHP1* silencing on MIA biosynthesis and JA pathway gene expression in *C. roseus* leaves. (**A**) Relative expression levels of *CrLHP1*, MIA biosynthetic genes and JA-responding genes in *CrLHP1*-silenced leaves. (**B**) The accumulation of catharanthine in *CrLHP1*-silenced leaves. (**C**) The accumulation of vindoline in *CrLHP1*-silenced leaves. qRT-PCR results were normalized to *CrActin1* expression and expressed relative to the baseline level in the empty vector control leaves. Standard errors, calculated from 3 independent biological replicates (each consisting of 10 leaves collected from 10 plants of similar size and developmental stage), are denoted by error bars. Statistical significance was determined using a Student’s *t*-test (*: *p* < 0.05).

To further elucidate the regulatory function of *CrLHP1* within the JA signaling cascade, we silenced its expression in *C. roseus* leaves using a TRV-based VIGS system. Compared to the pTRV2 empty vector control, suppression of *CrLHP1* significantly upregulated the transcript levels of established JA-responsive genes, including *CrJAR1*, *CrJR3*, and *CrAOC*. These results indicated that *CrLHP1* functions as a negative regulator of JA signaling. We next evaluated the impact of *CrLHP1* suppression on the downstream MIA biosynthetic pathway. The results showed that silencing *CrLHP1* led to a significant induction of key MIA biosynthetic genes, including *CrTDC*, *CrSGD*, and *CrG10H* (Figure 5A). Subsequent HPLC analysis demonstrated that this transcriptional reprogramming resulted in modified alkaloid profiles, where catharanthine content increased 1.37-fold relative to the control, while vindoline levels exhibited no statistically significant variation (Figure 5B). Collectively, these findings demonstrate that CrLHP1 might act as a critical repressor bridging the JA signaling cascade and specialized metabolism, effectively constraining MIA biosynthesis and alkaloid accumulation.

### 3.5. Structural Prediction and In Vitro Verification of the CrJAZ1-CrLHP1 Interaction

**Figure 6:**
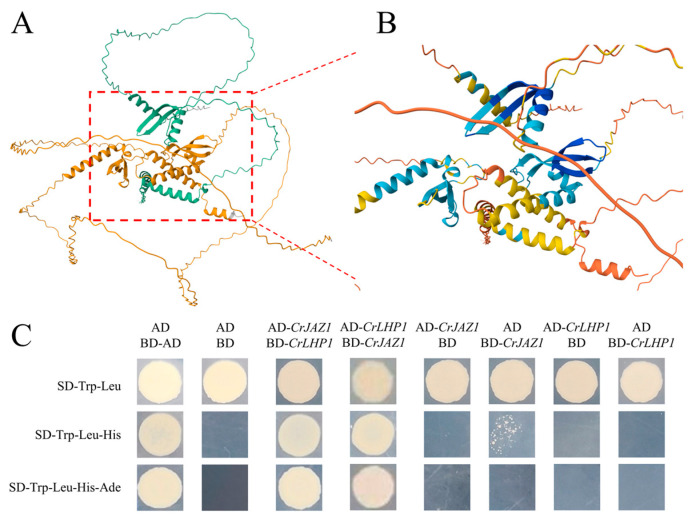
Alphafold-predicted and Y2H-verified interaction between CrJAZ1 and CrLHP1. (**A**) Overall structural view of the AlphaFold-predicted CrJAZ1-CrLHP1 interaction complex. The green model represents the CrJAZ1 protein, and the yellow model represents CrLHP1. (**B**) Close-up view of the CrJAZ1-CrLHP1 interaction interface with per-residue confidence. This panel highlights key regions of the predicted protein–protein interaction interface. The coloring scheme is based on AlphaFold’s predicted pLDDT values. Residues within the predicted interaction interface predominantly displayed medium to high confidence (light blue/yellow). (**C**) Validation of CrJAZ1-CrLHP1 interaction by Y2H. Note: AD, pGADT7; BD, pGBKT7; AD-*CrJAZ1*, pGADT7-*CrJAZ1*; BD-*CrJAZ1*, pGBKT7-*CrJAZ1*; AD-*CrLHP1*, pGADT7-*CrLHP1*; BD-*CrLHP1*, pGBKT7-*CrLHP1*; BD-AD, positive control.

Considering that JAZ proteins typically recruit the Polycomb Group (PcG) factor LHP1 to repress JA-responsive loci, we employed the deep learning algorithm AlphaFold to model the potential interaction between CrLHP1 and JAZ proteins in *C. roseus* (Table S3). The structural model generated by AlphaFold predicted spatial complementarity at the potential interaction interface between CrLHP1 and CrJAZ1. In addition, the complex exhibited a high interaction confidence score, suggesting a possible stable interaction (Figure 6A,B). We subsequently validated this in silico prediction using GAL4-based yeast two-hybrid (Y2H) assay. Co-expression of *CrLHP1* and *CrJAZ1* enabled robust yeast growth on stringent quadruple dropout medium (SD/-Trp/-Leu/-His/-Ade), verifying their specific binding. This stable interaction was consistently observed in reciprocal bait-prey configurations, with no autoactivation detected in the corresponding negative controls (Figure 6C). Collectively, these results confirm the direct physical association between CrLHP1 and CrJAZ1, suggesting a functional link between LHP1 and the JA signaling pathway. Further investigation of the CrLHP1-CrJAZ1 interaction in *C. roseus* will be important to strengthen this finding and to clarify their interaction dynamics in planta, particularly in response to JA signaling.

## 4. Discussion

The biosynthesis of MIAs in *C. roseus* is governed by a highly complex, spatio-temporal regulatory network [31,32]. While the transcriptional regulatory cascades controlling MIA biosynthesis have been extensively characterized, the epigenetic mechanisms fine-tuning this network remain largely elusive. Within the regulatory network of MIA biosynthesis, the JA signaling pathway has been established as a predominant regulatory factor [11]. The significance of this pathway stems from its fundamental role in plant defense and stress responses. As the primary phytohormone mediating plant immunity, JA coordinates broad-spectrum resistance by reprogramming cellular metabolism to produce toxic or deterrent phytochemicals. Consequently, JA signaling serves as a potent switch for specialized metabolic reprogramming, driving the large-scale synthesis of defensive metabolites essential for plant survival [12]. Specifically, the JA signaling cascade transcriptionally activates gene clusters encoding MIA biosynthetic enzymes, thereby promoting the robust accumulation of protective alkaloids [33,34]. In *C. roseus*, JA signaling has been demonstrated to directly regulate the expression of gene clusters involved in MIA biosynthesis, ultimately driving the synthesis and accumulation of corresponding defense metabolites [5,35,36,37]. Collectively, these findings indicate that the JA signaling pathway represents a fundamental adaptive strategy enabling plant survival under fluctuating environmental conditions [8].

JAZ proteins function as central and essential mediators in JA signaling in plants. The degradation of JAZ proteins relieves their repression of transcription factors, thereby activating the JA signaling cascade. This enables transcriptional reprogramming for defense responses and specialized metabolism. The released transcription factors subsequently bind to promoter regions of target genes. This binding initiates the expression of downstream genes, triggering diverse physiological processes regulated by JA signaling. These include defense activation, specialized metabolite biosynthesis, and adaptive growth modifications [18]. JAZ proteins exert inhibitory effects not only by sterically hindering transcription factors but also through the recruitment of corepressors such as the TPL/TPR family to establish stable transcriptional repression complexes [38]. This regulatory mechanism involves LHP1, which contributes to gene silencing through Polycomb group (PcG)-mediated pathways. Our genome-wide identification of 18 *LHP* family members reveals a substantial evolutionary expansion, particularly driven by tandem duplication events. The massive structural heterogeneity observed among these genes, ranging from simplified architectures featuring only the canonical Chromodomain (Motif 4) to highly complex, multi-motif configurations, strongly indicates significant evolutionary diversification and potential subfunctionalization. Despite this divergence, *CrLHP1* clusters tightly with *AtLHP1* and maintains strict conservation of the chromatin-binding domain, suggesting it has retained its fundamental core function as a possible epigenetic regulator within the *C. roseus* genome.

To further characterize the regulatory dynamics of MeJA-induced MIA biosynthesis, a range of concentrations was evaluated, revealing that 75 μM MeJA specifically upregulates key upstream biosynthetic genes such as *CrTDC*, *CrG10H*, *CrDL7H*, *CrLAMT*, and *CrSTR* while concomitantly suppressing the expression of *CrLHP1*. The physiological significance of *CrLHP1* is substantiated by our VIGS assay, which revealed that suppression of *CrLHP1* triggered the induction of JA responding genes (*CrJR3*, *CrAOC*, *CrJAR1*) and key MIA biosynthetic genes (*CrTDC*, *CrSGD*, *CrG10H*), resulting in a significant 1.37-fold increase in catharanthine accumulation. These findings raise the possibility that CrLHP1 could act as a negative regulator of MIA biosynthesis, potentially by maintaining a repressive chromatin landscape at certain target loci, which might thereby prevent the constitutive and energetically costly activation of defense responses and specialized metabolism. The alleviation of this possible epigenetic constraint likely reprograms the MIA metabolic network by redirecting metabolic flux toward the accumulation of specific alkaloids, although silencing *CrLHP1* yielded no significant impact on vindoline levels (Figure 5C). These findings suggest that CrLHP1 primarily targets and represses promoters within the catharanthine biosynthetic branch while potentially bypassing direct regulation of vindoline-specific downstream genes. Given that vindoline biosynthesis may be independently controlled by alternative mechanisms or specific transcription factors, the removal of CrLHP1-mediated repression alone is likely insufficient to activate the rate-limiting steps within the vindoline pathway.

The JA signaling cascade is the central orchestrator of plant defense responses and specialized metabolism, critically governing the biosynthesis of high-value MIAs [5]. As central components of this regulatory network, JAZ proteins function as master repressors to prevent the continuous and metabolically expensive activation of these pathways. Beyond acting as simple transcriptional repressors, JAZ proteins in *Arabidopsis* recruit the Polycomb Repressive Complex 2 component LHP1 to specific loci, thereby promoting the deposition and maintenance of repressive H3K27me3 modifications to ensure the sustained silencing of genes responsive to JA signaling [22]. The present study demonstrates that this epigenetic regulatory paradigm possibly extends to the medicinal plant *C. roseus*, as evidenced by structural complementarity predicted by AlphaFold3 and the robust physical interaction confirmed by yeast two-hybrid assays, which together indicate that CrJAZ1 directly binds to CrLHP1. The conservation of the JAZ-LHP1 interaction across phylogenetically diverse species raises the possibility that this partnership may represent a conserved regulatory module, potentially serving as a mechanistic link that couples phytohormone signaling to Polycomb group-mediated epigenetic silencing. This connection ensures that target downstream genes remain tightly repressed prior to the perception of environmental or developmental JA signals. By demonstrating that CrLHP1 acts as a possible chromatin regulator downstream of JAZ proteins, this study provides new mechanistic insights into how JA signaling controls the production of valuable pharmaceutical compounds at the chromatin level. This finding not only advances our understanding of species-specific regulation of specialized metabolism in medicinal plants but also offers potential new targets for metabolic engineering to enhance the production of anti-cancer alkaloids in *C. roseus*. Future research utilizing chromatin immunoprecipitation sequencing to track H3K27me3 deposition at MIA biosynthetic gene promoters will be essential to comprehensively map the epigenomic landscape mediated by this protein complex.

## Data Availability

Data will be provided on reasonable request to the corresponding author. The data are not publicly available due to the data also forming part of an ongoing study.

## Supplementary Materials

The following supporting information can be downloaded at: https://www.mdpi.com/article/10.3390/genes17050569/s1, Figure S1: Heatmap of Pearson correlation coefficients between candidate genes and MIA biosynthetic genes; Figure S2: PCR-amplification of *CrLHP1* (*KAI5647659.1*); Figure S3: Phylogenetic analysis and sequence alignment of JAZ orthologs in *C. roseus* and *A. thaliana*; Table S1: Primers for gene cloning and vector construction.; Table S2: Primers for gene expression analysis; Table S3: Predicted binding free energy of CrLHP1 protein with JAZ family members.
