# Supplementary material for: CrLHP1-CrJAZ1 Module Regulates Monoterpenoid Indole Alkaloid Biosynthesis via JA Signaling in Catharanthus roseus
Source: Genes (Basel). 2026 May 17;17(5):569. doi: 10.3390/genes17050569 (PMC13205712; doi:10.3390/genes17050569)
Supplement: Supplementary file 1 [file genes-17-00569-s001.zip › Figures S1-S3 .pdf]

Supplementary Information

CrLHP1-CrJAZ1 module regulates monoterpenoid indole alkaloid biosynthesis via JA signaling in Catharanthus roseus

Bingrun Yang [1,†], Wenhui Ma [1,†], Jianing Cheng [2], Xiaoxiao Gao [1,*] and Fang Yu [1,2,*]

1 School of Biological Engineering, Dalian Polytechnic University, Dalian, 116034, China

2 College of Bioscience and Biotechnology, Shenyang Agricultural University, Shenyang, 110866, China

† Contribute equally to this work.

* Correspondence: xxgao@dlpu.edu.cn (X.G.); fyu0506@gmail.com (F.Y.)

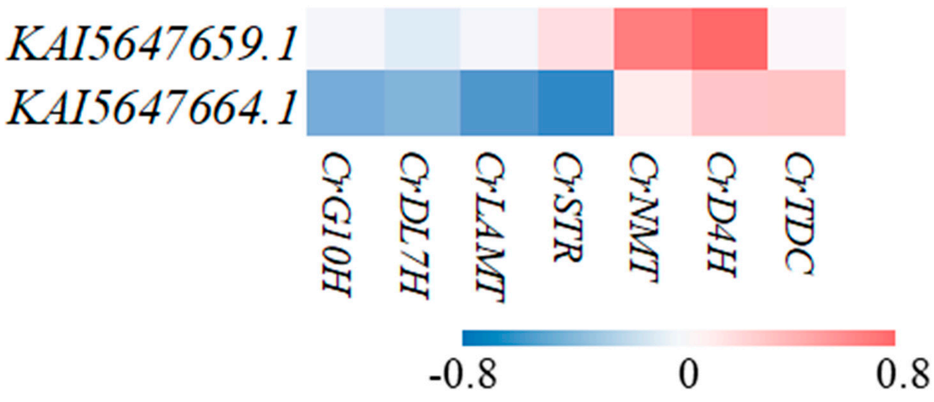

Figure S1. Heatmap of Pearson correlation coefficients between candidate genes and MIA biosynthetic genes. The x-axis indicates the MIA biosynthetic genes. The y-axis specifies the candidate genes (*KAI5647659.1* and *KAI5647664.1*). The color gradient denotes the magnitude of the Pearson correlation coefficients, with red signifying positive correlations and blue signifying negative correlations.

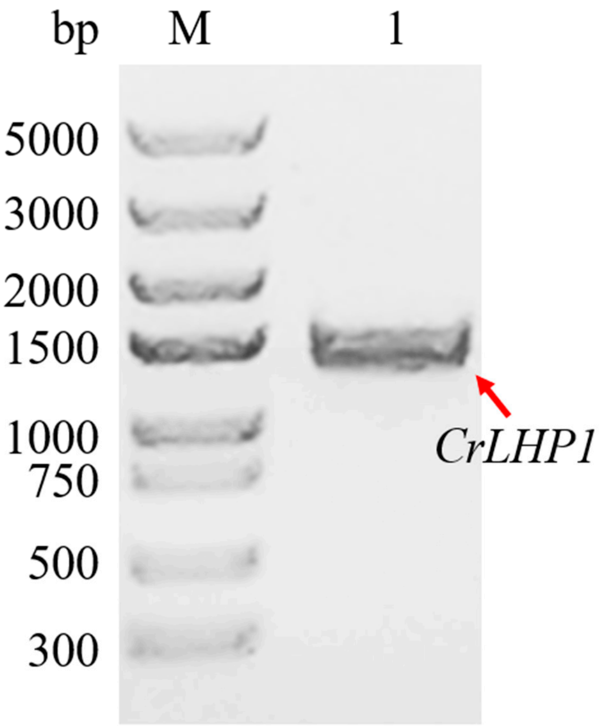

Figure S2. PCR-amplification of *CrLHP1* (*KAI5647659.1*). M: Molecular weight marker; Lane 1: amplified full-length *CrLHP1*.

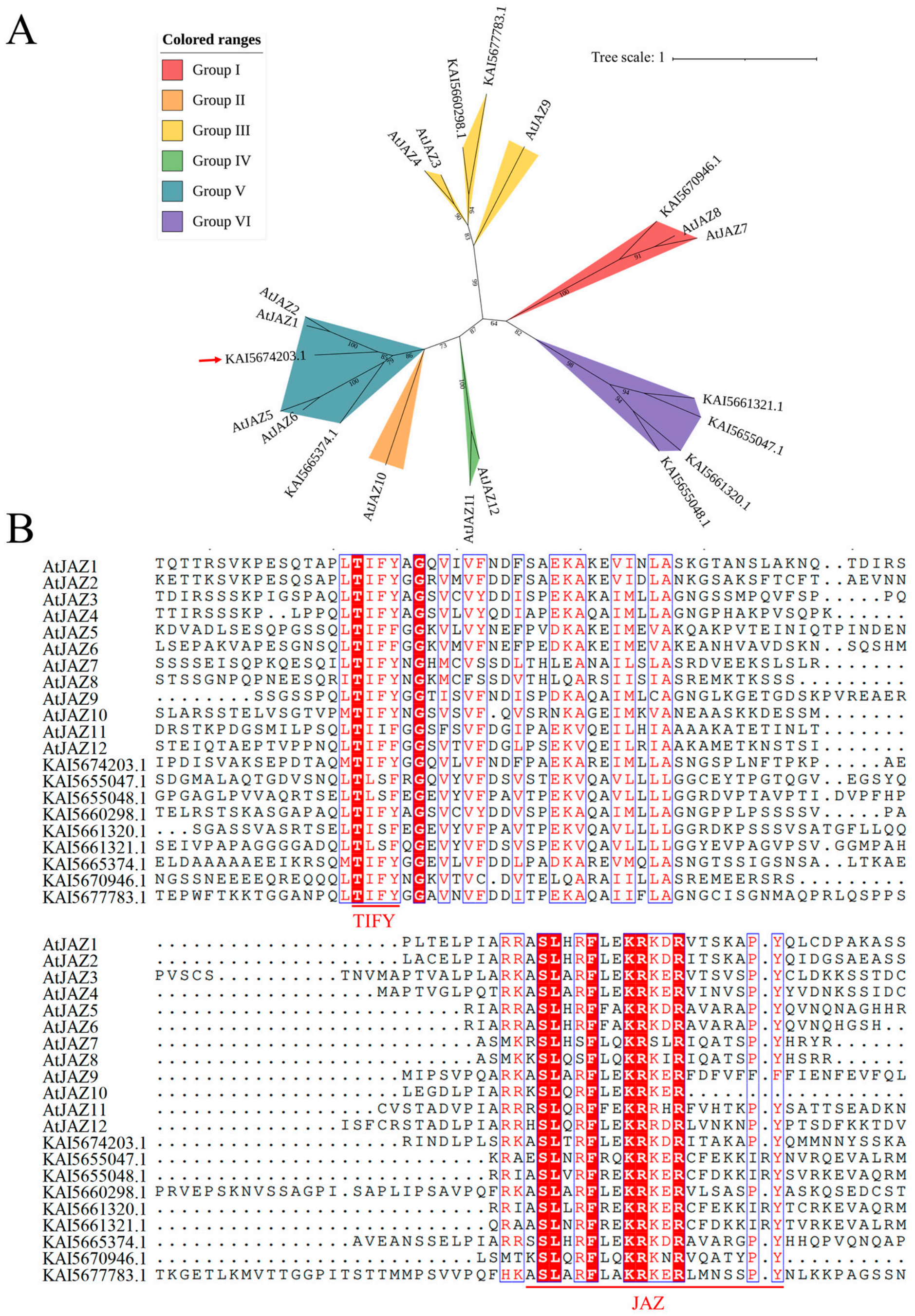

Figure S3. Phylogenetic analysis and sequence alignment of JAZ orthologs in *C. roseus* and *A. thaliana*. (A) Unrooted tree was constructed using the maximum likelihood method implemented in MEGA 11. The scale bar denotes the unit evolutionary distance, reflecting the number of substitutions per site accumulated along each branch. (B) Analysis of JAZ protein domain sequence alignment. Alignment results revealed conserved sequence features within the ZIM domain and JAZ domain of JAZ proteins from *C. roseus* and *A. thaliana*, as indicated within red lines.
